# Roles and functions of tumor-infiltrating lymphocytes and tertiary lymphoid structures in gastric cancer progression

**DOI:** 10.3389/fimmu.2025.1595070

**Published:** 2025-05-29

**Authors:** Zhiyuan Yao, Gengchen Li, Di Pan, Zichen Pei, Yan Fang, Haonan Liu, Zhengxiang Han

**Affiliations:** ^1^ Department of Oncology, The Affiliated Hospital of Xuzhou Medical College, Xuzhou, Jiangsu, China; ^2^ Department of Gastroenterology, The Affiliated Suzhou Hospital of Nanjing Medical University, Suzhou, Jiangsu, China

**Keywords:** gastric cancer, tertiary lymphoid structures, tumor-infiltrating lymphocytes, tumor microenvironment, immune checkpoint inhibitors, progression, biomarkers

## Abstract

Gastric cancer (GC), a leading cause of cancer mortality, exhibits profound molecular heterogeneity and immunosuppressive tumor microenvironment (TME) features that limit therapeutic efficacy. This review elucidates the dual roles of tertiary lymphoid structures (TLS) and tumor-infiltrating lymphocytes (TILs) in GC progression. TLS, ectopic lymphoid organs formed under chronic inflammation, correlate with improved survival and immunotherapy sensitivity by fostering effector T/B cell interactions and antigen presentation. Conversely, immunosuppressive TME components like regulatory T cells (Tregs) and tumor-associated macrophages (TAMs) drive immune evasion via cytokine-mediated suppression and checkpoint activation (PD-1/PD-L1). CD8^+^ T cells exert context-dependent effects, with high infiltration reducing recurrence risk but paradoxically inducing exhaustion in PD-L1-rich microenvironments. Th17 and memory T cells further modulate disease through IL-17-driven angiogenesis and CD45RO^+^ immune memory dynamics. Multi-omics-based TLS scoring and combinatorial therapies emerge as promising strategies to overcome resistance.

## 1 Introduction

Gastric cancer (GC) poses a significant public health challenge due to its substantial disease burden and clinical management complexities (1). Molecularly and phenotypically heterogeneous feature exhibits distinct clinical intervention strategies (2–4). The tumor microenvironment (TME) plays a pivotal role in therapeutic resistance (5–9), where emerging evidence highlights tertiary lymphoid structures (TLS) as critical immunological determinants. These ectopic lymphoid organs exhibit distinct morphogenesis and functional specialization compared to embryonically derived secondary lymphoid organs (SLOs). While SLOs develop constitutively during embryogenesis, TLS form *de novo* in inflamed non-lymphoid tissues via lymphoid neogenesis under chronic inflammatory conditions (10–12). Their molecular assembly is initiated by stromal CXCL13 and IL-7 secretion, recruiting lymphoid tissue inducer (LTi) cells that drive maturation through LTα/β and TNF signaling, ultimately inducing VEGF-C-dependent high endothelial venule (HEV) formation and PNAd-mediated lymphocyte homing (13, 14).

Notably, TLS are associated with prolonged OS and enhanced immunotherapy sensitivity in solid tumors (12, 15), likely through sustaining effector T-cell clonal expansion and promoting B-cell-mediated humoral immunity. This is particularly relevant given the current landscape of immune checkpoint inhibitors (ICIs), which reactivate antitumor immunity by targeting PD-1/PD-L1 signaling and offer survival benefits for heavily pretreated advanced patients (16). However, the objective response rate (ORR) in GC remains markedly lower than in immunogenic cancers such as melanoma (17–19), underscoring the need to better understand TLS biology and its clinical implications. Histological features including TLS maturity, spatial distribution patterns of tumor-infiltrating lymphocytes, and stromal composition are now recognized as critical biomarkers for predicting immunotherapy responsiveness (20–23), positioning TLS as both biological regulators and therapeutic targets in GC management.

## 2 Immunoregulatory network of TLS in GC

### 2.1 Spatial interplay between TLS and immune cells

GC-associated TLS exhibit a spatially organized immune cell architecture: B-cell zones are dominated by CD20^+^ B lymphocytes accompanied by CD21^+^ follicular dendritic cells, while T-cell zones primarily comprise CD3^+^ T lymphocytes, including CD8^+^ cytotoxic T lymphocytes (CTLs) and CD4^+^ helper T cells, forming a functionally complementary immune microenvironment (24–26). Notably, tumor-infiltrating B cells in GC predominantly cluster within TLS. These antigen-experienced B cells can differentiate into antigen-presenting cells, promoting CTL clonal expansion and survival by presenting tumor-associated antigens, thus serving as pivotal orchestrators of antitumor immunity (27). Clinico-pathological evidence reveals that TLS-high gastric tumors exhibit significantly elevated infiltration of CD20^+^ B cells, CD8^+^ T cells, and CD3^+^ T cells compared to TLS-low counterparts, with CD20^+^ B/CD8^+^ T cell co-infiltration independently correlated with prolonged overall survival (28, 29). Mechanistically, TLS enhance T-cell-mediated antitumor immunity by supporting CD8^+^ T cell differentiation into effector memory T cells and driving Th1-type cytokine secretion, like IFN-γ, TNF-α, as demonstrated by transcriptional profiling of tumor-infiltrating lymphocytes (30). Hennequin et al. (31) further identified a strong positive correlation between B-cell aggregate density within gastric TLS and Tbet^+^ effector T-cell infiltration, a phenotype linked to improved recurrence-free survival. These findings collectively suggest that TLS orchestrate T/B cell crosstalk to maintain tumor-immune equilibrium. Recent evidence reveals the spatiotemporal co-evolution of TLS and TILs during tumor progression, wherein immature TLS mature into organized FDC networks with HEVs (32, 33), while TILs transition from naïve/effector to memory/exhausted phenotypes, with longitudinal studies showing that TLS germinal center maturation correlates with CD8^+^ T cell proliferation but also eventual exhaustion, underscoring their dynamic interplay in antitumor immunity (34).

### 2.2 Prognostic significance of TLS and the immunosuppressive microenvironment

TLS development proceeds through distinct stages: (i) stromal cells secrete homeostatic chemokines to attract lymphocytes; (ii) lymphoid tissue inducer (LTi) cells are recruited to the inflammatory site; (iii) LTβR and TNF signaling drive vascular remodeling and lymphocyte retention; (iv) high endothelial venules (HEVs) expressing PNAd emerge, facilitating lymphocyte trafficking; and (v) organized B/T cell zones form with follicular dendritic cell (FDC) networks, establishing functional TLS capable of antigen presentation and immune cell activation (27, 35). The prognostic impact of TLS is critically modulated by TAMs. CD68^+^ TAM infiltration inversely correlates with TLS density in gastric cancer, while elevated TAM levels predict increased risks of tumor progression (36–40). Furthermore, immune checkpoint dysregulation within TLS may counteract their protective functions: high expression of TIGIT in TLS-associated CD20^+^ B cells accelerates CD8^+^ T cell exhaustion, correlating with reduced median OS. Intriguingly, this TIGIT-enriched subset may derive therapeutic benefit from adjuvant chemotherapy (41). In the gastric cancer microenvironment, TAMs polarize into M1-like (pro-inflammatory) or M2-like (immunosuppressive) phenotypes via cytokine/growth factor signals, regulating TLS formation (42). Mature TLS correlate with robust antitumor immunity, CD8^+^/memory T cell infiltration, and improved survival, supporting immune surveillance (43, 44). Immature TLS lack structured T/B zones/FDC networks, yielding poor responses. Mature TLS predict better immunotherapy efficacy via efficient neoantigen presentation/T cell priming, unlike immature TLS.

### 2.3 TLS and neoantigen-driven immune responses

Genomic studies have established a positive correlation between TLS formation and tumor neoantigen burden (45). As central hubs for antigen presentation within the TME, TLS facilitate cross-presentation of tumor-derived neoantigens via enriched mDCs, thereby driving T-cell receptor clonal expansion—a process validated in both gastric and other solid tumors (40, 46, 47). Single-cell technologies have unveiled the intricate connections among diverse cells within the complex tumor microenvironment (48), including novel cell subtypes (49, 50). Single-cell RNA sequencing analyses of GC-associated mTLS reveal mucosal-associated lymphoid tissue-derived IgA^+^ plasma cells and natural killer T (NKT) cell subsets, with upregulated complement activation-related genes, such as C1QA, C3AR1, suggesting coordinated humoral and innate antitumor mechanisms (51). Furthermore, Zhu et al. (52) demonstrated that TLS-resident naïve T cells undergo clonal selection and activation through direct contact with mDCs in lung cancer models. The homology of this mechanism in GC, however, requires experimental validation to confirm its conservation across malignancies.

### 2.4 TLS and cancer immunotherapy

TLS serve as pivotal immunotherapeutic hubs, with their biological traits correlating multidimensionally with treatment sensitivity (53, 54). Cottrell et al. (55) demonstrated TLS-plasma cell co-localization in PD-L1-responsive tumors, implicating TLS in enhancing effector T/B cell synergy. Memory B cells within TLS exhibit dual roles: as APCs driving T-cell expansion and as antibody-secreting plasma cells, while shaping pro-inflammatory microenvironments via TNF/IL-6/IFN-γ secretion (56). TLS density correlates with immune-active tumor microenvironments, supporting TLS-induction therapies (57). Genomically, TLS are enriched in EBV-positive, MSI-high, and PI3K-mutant gastric cancers, where TLR/NF-κB activation driven by high neoantigen loads may enhance responses to PD-1 blockade (28, 45, 58). Clinically, TLS scoring systems have emerged as valuable predictors of immunotherapy efficacy, with a 2.1-fold increase in objective response rate (ORR) observed in high-TLS patients, particularly those harboring CD103^+^ tissue-resident memory T cell niches (59, 60). Besides, current therapeutic approaches involving triggering the formation of TLS are being applied in GC therapy (61). Therapeutic strategies targeting TLS or checkpoint pathways differ in both mechanism and maturity of evidence. PI3K inhibitors (BAY1082439) promote TLS maturation through chemokine induction and HEV formation—findings supported by preclinical models and early-phase clinical data (62). TLR/NF-κB agonists (CpG-ODNs) stimulate *de novo* TLS formation and remain in the experimental phase with robust murine evidence but limited clinical translation (63, 64). Conversely, PD-1/PD-L1 inhibitors (pembrolizumab) primarily reverse T-cell exhaustion, with efficacy validated in phase II/III trials such as KEYNOTE-061 (65). Combination strategies (PI3K+PD-1 blockade) are currently under early-phase investigation and exhibit synergistic immunostimulatory effects in gastric and other solid tumors (66–68).

Notably, despite its promise as a predictive biomarker, the utility of TLS scoring techniques is constrained by several limitations. First, spatial heterogeneity within tumors means TLS density may vary significantly across sampled regions, leading to under- or overestimation depending on biopsy location. Second, sampling bias in endoscopic or surgical specimens may fail to capture peritumoral TLS clusters that critically influence immune responses. Third, TLS undergo dynamic remodeling during disease progression and treatment, including transitions from immature to mature states or regression following chemotherapy, complicating longitudinal assessments.

## 3 Role of TILs in gastric cancer development

### 3.1 Dynamics and functions of CD4^+^ and CD8^+^ T cells in gastric cancer

CD4^+^ helper T cell polarization imbalance drives GC immune evasion, with Th1/Th2 disequilibrium being pivotal. Th1 cells enhance cellular immunity via IL-2/IFN-γ/TNF-β, promoting CTL/NK activity, while Th2 cells stimulate humoral immunity through IL-4/IL-6/IL-10-mediated B-cell differentiation (69). Cross-regulation occurs via IFN-γ suppression of Th2 and Th2 cytokine inhibition of Th1 (70–74). *H. pylori* and dietary carcinogens promote Th2 bias, inducing Treg/M2 macrophage-mediated immunosuppressio*n* (75, 76). Th17 cells, regulated by TGF-β/IL-6/STAT3, secrete IL-17 to promote tumor progression primarily through pro-inflammatory and pro-angiogenic effects. IL-17 enhances angiogenesis by stimulating the expression of chemokines, while simultaneously recruiting neutrophils via IL-8/IL-17 feedback loops, thereby facilitating local inflammation and metastasis in gastric cancer (77). In early-stage GC, IL-17A can activate the NF-κB pathway and induce stromal remodeling, leading to enhanced tumor proliferation. Moreover, certain IL-17 cytokines, including IL-17B, IL-17C, and IL-17F, upregulate VEGF and MMP-9, contributing to vascular invasion and extracellular matrix degradation (78, 79).

Conversely, Th17 cells also exhibit immunostimulatory functions. IL-21 produced by Th17 cells recruits CD8+ cytotoxic T lymphocytes through the CXCR3–CXCL10 axis and enhances their cytolytic capacity by upregulating granzyme B expression. Furthermore, RORγt^+^ IL-17A^+^ Th17 cells are associated with increased infiltration of mast cells and NK cells, correlating with improved patient survival and suppression of M2 macrophage-mediated immunosuppression (78, 80). The duality of Th17 cell function appears to be context-dependent: promoting tumorigenesis via NF-κB in early-stage cancer while enhancing immunotherapy response in advanced disease (74, 76). Additionally, IL-17D and IL-17E have been shown to stimulate IFN-γ production by CD8+ T cells, contributing to antitumor immunity (78).

**Figure 1:**
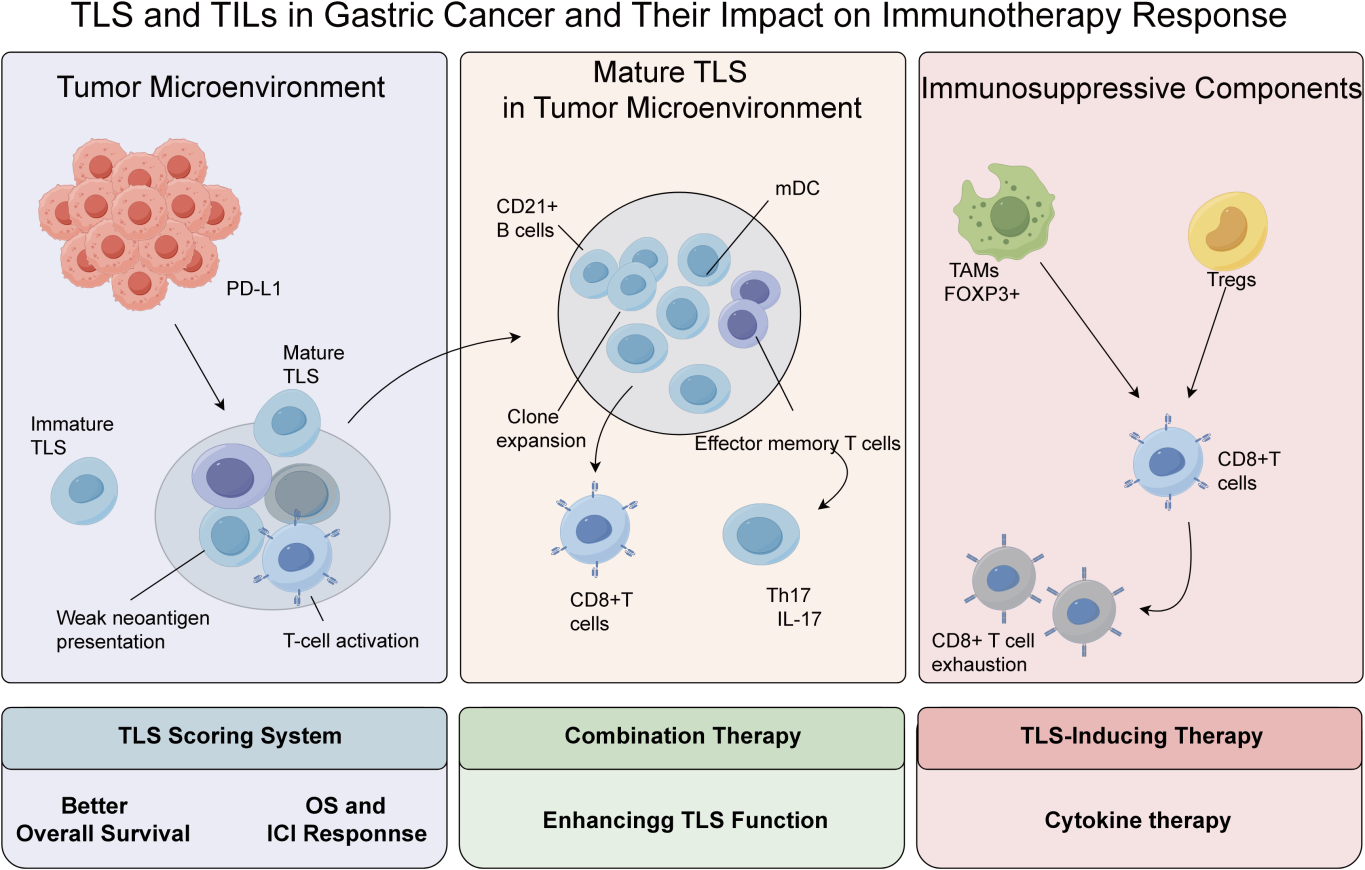
The immune interactions between TLS and TILs in gastric cancer and their impact on immunotherapy response.

CD8^+^ T cells, as central effectors of cellular immunity, mediate tumor cell lysis via MHC-I-restricted antigen recognition, migrating along chemokine gradients to infiltrate tumor parenchyma and executing cytotoxicity through granzyme-perforin-mediated exocytosis and Fas/FasL death receptor signaling (81). Pan-cancer analyses substantiate the prognostic universality of CD8^+^ T cell infiltration, with elevated densities correlating significantly with improved outcomes in cervical, colorectal, and breast cancers (82–84). In GC-specific studies, Li et al. (33) demonstrated that high CD8^+^ T cell infiltration inversely associates with histological grade (G1/G2) and early TNM staging (I/II), confirming their tumor-suppressive role. Furthermore, Wang et al. (85) revealed in multicohort analyses that GC patients with CD8^+^ T cell-rich infiltrates exhibit significantly reduced lymphovascular invasion and perineural infiltration rates, underscoring their metastasis-inhibitory potential. The immunosuppressive TME, however, counteracts these advantages: dysregulated PD-L1 upregulation engages the PD-1/PD-L1 inhibitory pathway, driving CD8^+^ T cells toward functional impairment and clonal depletion, a process strongly associated with aggressive disease manifestations (81). Thus, although the extent of CD8^+^ T cell infiltration provides essential prognostic insights in GC, their cytotoxic potential is continuously shaped by immunoediting processes within the tumor (**Figure 1**).

### 3.2 Regulatory T cells in gastric cancer pathogenesis and progression

Regulatory T cells (Tregs) play dual roles in gastric cancer, suppressing immunity via contact-dependent mechanisms and inhibitory IL-10 and TGF-β, while impairing NK cell activity (86–90). Treg subsets include CD4^+^CD25^+^, Tr1 (IL-10^+^), Th3 (TGF-β^+^), and CD4^−^CD8^−^ populations, with FOXP3 as the definitive marker for functional CD4^+^CD25^+^ Tregs (88). Clinically, FOXP3^+^ Treg infiltration correlates with advanced tumor stage and reduces 5-year survival by 41.3% (91). Notably, aberrant FOXP3 expression in GC cells promotes PBMC differentiation into Tregs via the miR-155/miR-21 axis, while inducing PBMC secretion of IL-35 and TGF-β to establish immune escape circuits (92). Spatial transcriptomic evidence demonstrates that FOXP3^+^ Tregs form immunosuppressive synapses with CD20^+^ B cells in metastatic niches, driving regulatory B cell (Breg) differentiation via LAG-3/MHC-II interactions to synergistically enforce immune tolerance (92–94). Recent studies have also identified a subset of CCR8^+^ tumor-specific Tregs in advanced GC exhibits strong immunosuppression and TLS proximity, impairing antitumor immunity by suppressing T-cell activation (95). Thus, targeting CCR8 may restore immune responses in treatment-resistant cases.

### 3.3 CD45RO^+^ memory T cells in gastric cancer pathogenesis and progression

CD45RO^+^ T cells, the core subset of memory T cells, play pivotal roles in antitumor immunity through their unique immune memory retention and effector cell activation properties. They express high levels of adhesion molecules like CD44, facilitating rapid inflammatory homing and endothelial adhesion, sustaining long-term memory and swift secondary immune responses. Clinically, CD45RO^+^ T cells decline during GC progression: stage III–IV patients show reduced peripheral frequencies versus healthy controls and stage I–II patients, with metastatic tumors exhibiting lower CD45RO^+^ density than non-invasive lesions. This depletion impairs tumor-specific T cell activation, hindering metastatic cell clearance (96). Mechanistically, CD45RO^+^ memory T cell decline may stem from chronic tumor-associated antigen exposure-induced clonal exhaustion, compounded by excessive TGF-β secretion in the TME that disrupts memory-to-effector differentiation, ultimately fostering a pro-metastatic immune landscape.

## 4 Impact of tumor-infiltrating lymphocytes on gastric cancer prognosis

### 4.1 Prognostic implications of CD4^+^ T cells in gastric cancer

The dynamic equilibrium of CD4^+^ T cell subsets, particularly the Th1/Th2 polarization bias, holds critical prognostic significance in GC immunoregulation. Th1 cells orchestrate antitumor immunity through IL-2 and IFN-γ secretion, activating CTLs and enhancing NK cell cytotoxicity, whereas Th2 cells drive humoral responses via IL-4, IL-6, and IL-10 production, with reciprocal regulatory mechanisms maintaining immune homeostasis (97). GC patients exhibit characteristic Th1 suppression. Th2 activation, a disequilibrium that impairs immune surveillance and accelerates disease progression (98). Clinical evidence indicates that Th2-dominant polarization correlates with elevated risks of tumor recurrence and metastasis, mechanistically linked to Treg expansion and M2-polarized TAM infiltration. Consequently, contemporary immunotherapeutic strategies focus on redirecting naïve T cell differentiation toward Th1 polarization to restore Th1/Th2 balance, while monitoring IL-2/IL-4 cytokine profiles in tumor tissues provides molecular insights for personalized treatment optimization and prognostic stratification.

### 4.2 Impact of regulatory T cells on GC prognosis

Regulatory T cells (Tregs), as pivotal immunosuppressive components within the TME, exert profound clinical significance in GC prognosis through dynamic functional modulation. Inflammatory factors play important roles in disease’s progression (99–103). Tregs impair antitumor immunity by secreting inhibitory cytokines and inducing effector T cell dysfunction via direct contact-dependent mechanisms such as CTLA-4/B7-1 interactions, while concurrently promoting vascular endothelial apoptosis to facilitate immune escape (104). Notably, advanced GC patients exhibit characteristic peripheral Treg expansion coupled with B-cell lymphopenia, an immune imbalance partially reversible through neoadjuvant chemotherapy combined with surgical intervention, which restores B-cell-mediated humoral immunity and improves survival outcomes. The prognostic relevance of Tregs has galvanized efforts to develop novel therapeutic strategies targeting the FOXP3 signaling axis, aiming to deplete Tregs or disrupt their immunosuppressive functions to remodel the TME, thereby advancing precision immunotherapy for metastatic GC (105).

### 4.3 Prognostic impact of CD45RO^+^ memory T cells in GC

CD45RO^+^ memory T cells, a critical subset of TILs, exhibit stage-dependent prognostic associations with GC progression. In early-stage GC, CD45RO^+^ cells confer physiological protection by sustaining immune surveillance, with infiltration density positively correlating with tumor cell clearance efficiency. Conversely, in advanced-stage patients, high CD45RO^+^ infiltration significantly correlates with improved disease-free survival (DFS) and OS, underscoring its phase-specific prognostic utility (106–108). CD45RO^+^ T cell infiltration is an independent predictor of survival benefit in advanced solid tumors (106, 107). Notably, while post-operative CD45RO^+^ infiltration serves as a biomarker for prognostic stratification in advanced GC, its predictive power lacks statistical significance in early-stage disease, likely attributable to incomplete activation of immunoediting mechanisms during initial tumor evolution (109). Clinical observations reveal markedly reduced peripheral CD45RO^+^ T cell proportions in recurrent/metastatic patients, though surgical resection combined with adjuvant therapy can partially reverse this immune exhaustion phenotype by restoring effector T cell cytotoxicity. Despite the therapeutic potential of targeting CD45RO^+^ cell dynamics, the development of specific regulatory strategies and pharmacological interventions necessitates further elucidation through multi-omics.

### 4.4 Prognostic impact of CD8^+^ T cells in GC

Immune cells, as core effector components of the TME (110–114), exhibit complex spatial distribution patterns that critically influence cancer outcomes (115–117). High-density CD8^+^ T cell infiltration reduces recurrence risk and improves OS through granzyme-perforin-mediated cytotoxicity and Fas/FasL death receptor signaling, enabling tumor cell-specific elimination (118). demonstrated via multivariate Cox regression analysis of a 509-patient GC cohort that CD8^+^ T cell density serves as an independent prognostic factor for OS, showing a strong positive correlation with survival benefit. Paradoxically, Thompson et al. (119) revealed that increased CD8^+^ T cell infiltration may activate the PD-L1/PD-1 immune checkpoint axis, inducing T cell exhaustion and shortening OS. Notably, recent meta-analyses consolidate evidence supporting the unequivocal protective role of CD8^+^ T cells in GC prognosis, though their effect magnitude is modulated by tumor molecular subtypes and therapeutic regimens (120). Thus, combining immune modulation strategies to enhance CD8^+^ T cell infiltration with PD-1/PD-L1 blockade to counteract functional suppression represents a promising therapeutic paradigm for optimizing multimodal GC treatment efficacy (**Table 1**).

**Table 1:** Tumor-infiltrating lymphocyte (TIL) subsets in gastric cancer: functional dichotomy and clinical impact.

TIL Subset	Pro-Tumor Mechanisms	Anti-Tumor Mechanisms	Prognostic Association	Targeted Strategies
CD8+ T cells	PD-L1-induced exhaustion via TIM-3/LAG-3 upregulation	Granzyme B-mediated cytotoxicity; IFN-γ-dependent MHC-I upregulation	High infiltration improves OS (HR=0.54) in PD-L1-low tumors	PD-1 blockade + IL-15 superagonist reverses exhaustion
​Th17 cells	IL-17A-driven angiogenesis (VEGF↑, CXCL8↑)	IL-21-mediated CTL recruitment via CXCL10	Dual role: Early-stage IL-17A↑ predicts poor OS; Late-stage correlates with ICI response	Anti-IL-17A mAbs in early GC; IL-17F agonists in advanced
​Tregs	FOXP3+CTLA-4+ subset induces CD8+ anergy via TGF-β/IL-35	Tumor-intrinsic FOXP3 suppresses proliferation	≥50 FOXP3+ cells/HPF reduces 5-year OS by 41.3%	Depleting CCR4+ Tregs using mogamulizumab
​CD45RO+ Tm	Chronic antigen exposure induces clonal attrition	Immune memory against tumor stem cells via CD103+ TRM	Post-op CD45RO+ density ≥25% predicts 68% 3-year DFS	Personalized vaccines to expand tumor-specific Tm
​Bregs	LAG-3+ Bregs secrete IL-10; Promote TAM M2 polarization	TLS-associated B cells enhance PD-1 inhibitor efficacy	Breg/Tfh ratio >2.5 correlates with peritoneal metastasis	CD40 agonists + BTK inhibitors to reprogram B cell fate

## 5 Conclusion

The interplay between TLS, TIL subsets, and immunosuppressive networks defines GC progression and therapeutic outcomes. TLS enhance antitumor immunity via lymphoid neogenesis and neoantigen presentation, yet their efficacy is counterbalanced by Treg/TAM-mediated suppression and checkpoint dysregulation. CD8^+^ T cells and Th17 subsets exhibit dual roles, influenced by molecular subtypes and TME spatial architecture. Memory T cell attrition and Th1/Th2 imbalance further impair immune surveillance, highlighting the need for stage-specific therapeutic approaches. Single-cell profiling of TIL exhaustion states and spatial-temporal TLS maturation analysis can refine prognostic and therapeutic strategies (121–123). Key TLS biomarkers such as CXCL13/CCL21 levels and Tfh/B cell clonality may improve patient stratification for combination immunotherapies. Future research should focus on TLS induction, T cell rejuvenation, and biomarker-driven combinations. Cross-omics validation and standardized TLS quantification are essential for clinical translation. Targeted delivery systems could enhance TLS-localized immunomodulation, minimizing systemic toxicity. These advances promise to bridge TLS biology with precision oncology, optimizing GC immunotherapy.

## References

[1] Sung H Ferlay J Siegel RL Laversanne M Soerjomataram I Jemal A. Global cancer statistics 2020: GLOBOCAN estimates of incidence and mortality worldwide for 36 cancers in 185 countries. CA Cancer J Clin. (2021) 71:209–49. doi: 10.3322/caac.21660 33538338

[2] Chen C Shao Y Ye C Yu X Hu M Yan J. Weighted gene coexpression network analysis identifies neutrophil-related molecular subtypes and their clinical significance in gastric cancer. Cancer Manag Res. (2025) 17:397–418. doi: 10.2147/CMAR.S500215 40040634 PMC11878151

[3] Yan J Ye G Jin Y Miao M Li Q Zhou H. Identification of novel prognostic circRNA biomarkers in circRNA-miRNA-mRNA regulatory network in gastric cancer and immune infiltration analysis. BMC Genomics. (2023) 24:323. doi: 10.1186/s12864-023-09421-2 37312060 PMC10262520

[4] Zhu H Zhao Y Wang Y Wei G Liu J. Understanding the relationship between cuproptosis and the development of hepatocellular carcinoma: implications for targeted therapies. Front Immunol. (2025) 16:1557223. doi: 10.3389/fimmu.2025.1557223 40145101 PMC11936877

[5] Yu X Shao Y Dong H Yan J Zhang X Ye G. Molecular subtype of gastric cancer based on apoptosis-related genes reveals differential immune microenvironment and intratumoral microorganisms distribution. BMC Cancer. (2025) 25:12. doi: 10.1186/s12885-024-13411-2 39762768 PMC11702164

[6] Deng Y Shi M Yi L Naveed Khan M Xia Z Li X. Eliminating a barrier: Aiming at VISTA, reversing MDSC-mediated T cell suppression in the tumor microenvironment. Heliyon. (2024) 10:e37060. doi: 10.1016/j.heliyon.2024.e37060 39286218 PMC11402941

[7] Gong X Chi H Strohmer DF Teichmann AT Xia Z Wang Q. Exosomes: A potential tool for immunotherapy of ovarian cancer. Front Immunol. (2022) 13:1089410. doi: 10.3389/fimmu.2022.1089410 36741380 PMC9889675

[8] Zhao Y Wei K Chi H Xia Z Li X. IL-7: A promising adjuvant ensuring effective T cell responses and memory in combination with cancer vaccines? Front Immunol. (2022) 13:1022808. doi: 10.3389/fimmu.2022.1022808 36389666 PMC9650235

[9] Fang Z Shao Y Hu M Yan J Ye G. Biological roles and molecular mechanism of circular RNAs in epithelial-mesenchymal transition of gastrointestinal Malignancies. Oncol Res. (2025) 33:549–66. doi: 10.32604/or.2024.051589 PMC11915071 40109856

[10] Drayton DL Liao S Mounzer RH Ruddle NH. Lymphoid organ development: from ontogeny to neogenesis. Nat Immunol. (2006) 7:344–53. doi: 10.1038/ni1330 16550197

[11] Dieu-Nosjean MC Goc J Giraldo NA Sautès-Fridman C Fridman WH. Tertiary lymphoid structures in cancer and beyond. Trends Immunol. (2014) 35:571–80. doi: 10.1016/j.it.2014.09.006 25443495

[12] Dieu-Nosjean MC Giraldo NA Kaplon H Germain C Fridman WH Sautès-Fridman C. Tertiary lymphoid structures, drivers of the anti-tumor responses in human cancers. Immunol Rev. (2016) 271:260–75. doi: 10.1111/imr.12405"10.1111/imr.12405 27088920

[13] Martinet L Garrido I Filleron T Le Guellec S Bellard E Fournie JJ. Human solid tumors contain high endothelial venules: association with T- and B-lymphocyte infiltration and favorable prognosis in breast cancer. Cancer Res. (2011) 71:5678–87. doi: 10.1158/0008-5472.CAN-11-0431 21846823

[14] Jones GW Hill DG Jones SA. Understanding immune cells in tertiary lymphoid organ development: it is all starting to come together. Front Immunol. (2016) 7:401. doi: 10.3389/fimmu.2016.00401 27752256 PMC5046062

[15] Tang H Zhu M Qiao J Fu YX. Lymphotoxin signalling in tertiary lymphoid structures and immunotherapy. Cell Mol Immunol. (2017) 14:809–18. doi: 10.1038/cmi.2017.13 PMC5649108 28413217

[16] Jin W Yang Q Chi H Wei K Zhang P Zhao G. Ensemble deep learning enhanced with self-attention for predicting immunotherapeutic responses to cancers. Front Immunol. (2022) 13:1025330. doi: 10.3389/fimmu.2022.1025330 36532083 PMC9751999

[17] Fuchs CS Doi T Jang RW Muro K Satoh T MaChado M. Safety and efficacy of pembrolizumab monotherapy in patients with previously treated advanced gastric and gastroesophageal junction cancer: phase 2 clinical KEYNOTE-059 trial. JAMA Oncol. (2018) 4:e180013. doi: 10.1001/jamaoncol.2018.0013 29543932 PMC5885175

[18] Janjigian YY Bendell J Calvo E Kim JW Ascierto PA Sharma P. CheckMate-032 study: efficacy and safety of nivolumab and nivolumab plus ipilimumab in patients with metastatic esophagogastric cancer. J Clin Oncol. (2018) 36:2836–44. doi: 10.1200/JCO.2017.76.6212 PMC6161834 30110194

[19] Mahoney KM Rennert PD Freeman GJ. Combination cancer immunotherapy and new immunomodulatory targets. Nat Rev Drug Discov. (2015) 14:561–84. doi: 10.1038/nrd4591 26228759

[20] Ladányi A Kiss J Somlai B Gilde K Fejos Z Mohos A. Density of DC-LAMP(+) mature dendritic cells in combination with activated T lymphocytes infiltrating primary cutaneous melanoma is a strong independent prognostic factor. Cancer Immunol Immunother. (2007) 56:1459–69. doi: 10.1007/s00262-007-0286-3 PMC11030123 17279413

[21] Kroeger DR Milne K Nelson BH. Tumor-infiltrating plasma cells are associated with tertiary lymphoid structures, cytolytic T-cell responses, and superior prognosis in ovarian cancer. Clin Cancer Res. (2016) 22:3005–15. doi: 10.1158/1078-0432.CCR-15-2762 26763251

[22] Dieu-Nosjean MC Antoine M Danel C Heudes D Wislez M Poulot V. Long-term survival for patients with non-small-cell lung cancer with intratumoral lymphoid structures. J Clin Oncol. (2008) 26:4410–7. doi: 10.1200/JCO.2007.15.0284 18802153

[23] Di Caro G Bergomas F Grizzi F Doni A Bianchi P Malesci A. Occurrence of tertiary lymphoid tissue is associated with T-cell infiltration and predicts better prognosis in early-stage colorectal cancers. Clin Cancer Res. (2014) 20:2147–58. doi: 10.1158/1078-0432.CCR-13-2590 24523438

[24] Yamakoshi Y Tanaka H Sakimura C Deguchi S Mori T Tamura T. Immunological potential of tertiary lymphoid structures surrounding the primary tumor in gastric cancer. Int J Oncol. (2020) 57:171–82. doi: 10.3892/ijo.2020.5042 PMC7252463 32319601

[25] Petitprez F de Reynies A Keung EZ Chen TW Sun CM Calderaro J. B cells are associated with survival and immunotherapy response in sarcoma. Nature. (2020) 577:556–60. doi: 10.1038/s41586-019-1906-8 31942077

[26] Lund FE Randall TD. Effector and regulatory B cells: modulators of CD4+ T cell immunity. Nat Rev Immunol. (2010) 10:236–47. doi: 10.1038/nri2729 PMC3038334 20224569

[27] Sautès-Fridman C Petitprez F Calderaro J Fridman WH. Tertiary lymphoid structures in the era of cancer immunotherapy. Nat Rev Cancer. (2019) 19:307–25. doi: 10.1038/s41568-019-0144-6 31092904

[28] Yu JS Huang WB Zhang YH Chen J Li J Fu HF. The association of immune cell infiltration and prognostic value of tertiary lymphoid structures in gastric cancer. Neoplasma. (2022) 69:886–98. doi: 10.4149/neo_2022_220128N123 35603954

[29] Sakimura C Tanaka H Okuno T Hiramatsu S Muguruma K Hirakawa K. B cells in tertiary lymphoid structures are associated with favorable prognosis in gastric cancer. J Surg Res. (2017) 215:74–82. doi: 10.1016/j.jss.2017.03.033 28688665

[30] Marisa L Svrcek M Collura A Becht E Cervera P Wanherdrick K. The balance between cytotoxic T-cell lymphocytes and immune checkpoint expression in the prognosis of colon tumors. J Natl Cancer Inst. (2018) 110:10. doi: 10.1093/jnci/djx136 28922790

[31] Hennequin A Derangère V Boidot R Apetoh L Vincent J Orry D. Tumor infiltration by Tbet+ effector T cells and CD20+ B cells is associated with survival in gastric cancer patients. Oncoimmunology. (2016) 5:e1054598. doi: 10.1080/2162402X.2015.1054598 27057426 PMC4801425

[32] Shu DH Sidiropoulos DN. Maturation of tertiary lymphoid structures. Methods Mol Biol. (2025) 2864:43–55. doi: 10.1007/978-1-0716-4184-2_3 39527216

[33] Li Q Zhang D He W Chen T Yan Z Gao X. CD8(+) T cells located in tertiary lymphoid structures are associated with improved prognosis in patients with gastric cancer. Oncol Lett. (2020) 20:2655–64. doi: 10.3892/ol.2020.11828 PMC7400769 32782582

[34] Kasikova L Rakova J Hensler M Lanickova T Tomankova J Pasulka J. Tertiary lymphoid structures and B cells determine clinically relevant T cell phenotypes in ovarian cancer. Nat Commun. (2024) 15:2528. doi: 10.1038/s41467-024-46873-w 38514660 PMC10957872

[35] Carragher DM Rangel-Moreno J Randall TD. Ectopic lymphoid tissues and local immunity. Semin Immunol. (2008) 20:26–42. doi: 10.1016/j.smim.2007.12.004 18243731 PMC2276727

[36] Jeremiasen M Borg D Hedner C Svensson M Nodin B Leandersson K. Tumor-associated CD68(+), CD163(+), and MARCO(+) macrophages as prognostic biomarkers in patients with treatment-naïve gastroesophageal adenocarcinoma. Front Oncol. (2020) 10:534761. doi: 10.3389/fonc.2020.534761 33194593 PMC7645217

[37] He W Zhang D Liu H Chen T Xie J Peng L. The high level of tertiary lymphoid structure is correlated with superior survival in patients with advanced gastric cancer. Front Oncol. (2020) 10:980. doi: 10.3389/fonc.2020.00980 32733793 PMC7358602

[38] Liu F Huang J Liu X Cheng Q Luo C Liu Z. CTLA-4 correlates with immune and clinical characteristics of glioma. Cancer Cell Int. (2020) 20:7. doi: 10.1186/s12935-019-1085-6 31911758 PMC6945521

[39] Cheng N Li P Cheng H Zhao X Dong M Zhang Y. Prognostic value of tumor-infiltrating lymphocytes and tertiary lymphoid structures in epstein-barr virus-associated and -negative gastric carcinoma. Front Immunol. (2021) 12:692859. doi: 10.3389/fimmu.2021.692859 34276684 PMC8281029

[40] Goc J Germain C Vo-Bourgais TK Lupo A Klein C Knockaert S. Dendritic cells in tumor-associated tertiary lymphoid structures signal a Th1 cytotoxic immune contexture and license the positive prognostic value of infiltrating CD8+ T cells. Cancer Res. (2014) 74:705–15. doi: 10.1158/0008-5472.CAN-13-1342 24366885

[41] Liu H Wu J Xu X Wang H Zhang C Yin S. Peritumoral TIGIT(+)CD20(+) B cell infiltration indicates poor prognosis but favorable adjuvant chemotherapeutic response in gastric cancer. Int Immunopharmacol. (2022) 108:108735. doi: 10.1016/j.intimp.2022.108735 35405596

[42] Niu L Chen T Yang A Yan X Jin F Zheng A. Macrophages and tertiary lymphoid structures as indicators of prognosis and therapeutic response in cancer patients. Biochim Biophys Acta Rev Cancer. (2024) 1879:189125. doi: 10.1016/j.bbcan.2024.189125 38851437

[43] Sensi B Angelico R Toti L Conte L Coppola A Tisone G. Mechanism, potential, and concerns of immunotherapy for hepatocellular carcinoma and liver transplantation. Curr Mol Pharmacol. (2024) 17:e18761429310703. doi: 10.2174/0118761429310703240823045808 39225204

[44] Lin A Jiang A Huang L Li Y Zhang C Zhu L. From chaos to order: optimizing fecal microbiota transplantation for enhanced immune checkpoint inhibitors efficacy. Gut Microbes. (2025) 17:2452277. doi: 10.1080/19490976.2025.2452277 39826104 PMC12716052

[45] Lin Z Huang L Li S Gu J Cui X Zhou Y. Pan-cancer analysis of genomic properties and clinical outcome associated with tumor tertiary lymphoid structure. Sci Rep. (2020) 10:21530. doi: 10.1038/s41598-020-78560-3 33299035 PMC7725838

[46] Rooney MS Shukla SA Wu CJ Getz G Hacohen N. Molecular and genetic properties of tumors associated with local immune cytolytic activity. Cell. (2015) 160:48–61. doi: 10.1016/j.cell.2014.12.033 25594174 PMC4856474

[47] Brown SD Warren RL Gibb EA Martin SD Spinelli JJ Nelson BH. Neo-antigens predicted by tumor genome meta-analysis correlate with increased patient survival. Genome Res. (2014) 24:743–50. doi: 10.1101/gr.165985.113 PMC4009604 24782321

[48] Yan J Yu X Li Q Miao M Shao Y. Machine learning to establish three sphingolipid metabolism genes signature to characterize the immune landscape and prognosis of patients with gastric cancer. BMC Genomics. (2024) 25:319. doi: 10.1186/s12864-024-10243-z 38549047 PMC10976768

[49] Zhang P Pei S Wu L Xia Z Wang Q Huang X. Integrating multiple machine learning methods to construct glutamine metabolism-related signatures in lung adenocarcinoma. Front Endocrinol (Lausanne). (2023) 14:1196372. doi: 10.3389/fendo.2023.1196372 37265698 PMC10229769

[50] Liu J Zhang P Yang F Jiang K Sun S Xia Z. Integrating single-cell analysis and machine learning to create glycosylation-based gene signature for prognostic prediction of uveal melanoma. Front Endocrinol (Lausanne). (2023) 14:1163046. doi: 10.3389/fendo.2023.1163046 37033251 PMC10076776

[51] Jia L Wang T Zhao Y Zhang S Ba T Kuai X. Single-cell profiling of infiltrating B cells and tertiary lymphoid structures in the TME of gastric adenocarcinomas. Oncoimmunology. (2021) 10:1969767. doi: 10.1080/2162402X.2021.1969767 34513317 PMC8425751

[52] Zhu W Germain C Liu Z Sebastian Y Devi P Knockaert S. A high density of tertiary lymphoid structure B cells in lung tumors is associated with increased CD4(+) T cell receptor repertoire clonality. Oncoimmunology. (2015) 4:e1051922. doi: 10.1080/2162402X.2015.1051922 26587322 PMC4635865

[53] Xie Y Peng H Hu Y Jia K Yuan J Liu D. Immune microenvironment spatial landscapes of tertiary lymphoid structures in gastric cancer. BMC Med. (2025) 23:59. doi: 10.1186/s12916-025-03889-3 39901202 PMC11792408

[54] Fridman WH Meylan M Petitprez F Sun CM Italiano A Sautès-Fridman C. B cells and tertiary lymphoid structures as determinants of tumour immune contexture and clinical outcome. Nat Rev Clin Oncol. (2022) 19:441–57. doi: 10.1038/s41571-022-00619-z 35365796

[55] Cottrell TR Thompson ED Forde PM Stein JE Duffield AS Anagnostou V. Pathologic features of response to neoadjuvant anti-PD-1 in resected non-small-cell lung carcinoma: a proposal for quantitative immune-related pathologic response criteria (irPRC). Ann Oncol. (2018) 29:1853–60. doi: 10.1093/annonc/mdy218 PMC6096736 29982279

[56] Helmink BA Reddy SM Gao J Zhang S Basar R Thakur R. B cells and tertiary lymphoid structures promote immunotherapy response. Nature. (2020) 577:549–55. doi: 10.1038/s41586-019-1922-8 PMC8762581 31942075

[57] Cabrita R Lauss M Sanna A Donia M Skaarup Larsen M Mitra S. Tertiary lymphoid structures improve immunotherapy and survival in melanoma. Nature. (2020) 577:561–5. doi: 10.1038/s41586-019-1914-8 31942071

[58] Kim ST Cristescu R Bass AJ Kim KM Odegaard JI Kim K. Comprehensive molecular characterization of clinical responses to PD-1 inhibition in metastatic gastric cancer. Nat Med. (2018) 24:1449–58. doi: 10.1038/s41591-018-0101-z 30013197

[59] Jiang Q Tian C Wu H Min L Chen H Chen L. Tertiary lymphoid structure patterns predicted anti-PD1 therapeutic responses in gastric cancer. Chin J Cancer Res. (2022) 34:365–82. doi: 10.21147/j.issn.1000-9604.2022.04.05 PMC9468020 36199531

[60] Mori T Tanaka H Deguchi S Yamakoshi Y Miki Y Yoshii M. Clinical efficacy of nivolumab is associated with tertiary lymphoid structures in surgically resected primary tumors of recurrent gastric cancer. PloS One. (2022) 17:e0262455. doi: 10.1371/journal.pone.0262455 34995329 PMC8741034

[61] Chen W Zhang L Gao M Zhang N Wang R Liu Y. Role of tertiary lymphoid structures and B cells in clinical immunotherapy of gastric cancer. Front Immunol. (2024) 15:1519034. doi: 10.3389/fimmu.2024.1519034 39840050 PMC11747648

[62] Chen Y Wu Y Yan G Zhang G. Tertiary lymphoid structures in cancer: maturation and induction. Front Immunol. (2024) 15:1369626. doi: 10.3389/fimmu.2024.1369626 38690273 PMC11058640

[63] Gallotta M Assi H Degagné É Kannan SK Coffman RL Guiducci C. Inhaled TLR9 agonist renders lung tumors permissive to PD-1 blockade by promoting optimal CD4(+) and CD8(+) T-cell interplay. Cancer Res. (2018) 78:4943–56. doi: 10.1158/0008-5472.CAN-18-0729 29945961

[64] Torrejon DY Abril-Rodriguez G Champhekar AS Tsoi J Campbell KM Kalbasi A. Overcoming genetically based resistance mechanisms to PD-1 blockade. Cancer Discov. (2020) 10:1140–57. doi: 10.1158/2159-8290.CD-19-1409 PMC7416458 32467343

[65] Fuchs CS Özgüroğlu M Bang YJ Di Bartolomeo M Mandala M Ryu MH. Pembrolizumab versus paclitaxel for previously treated PD-L1-positive advanced gastric or gastroesophageal junction cancer: 2-year update of the randomized phase 3 KEYNOTE-061 trial. Gastric Cancer. (2022) 25:197–206. doi: 10.1007/s10120-021-01227-z 34468869 PMC8732941

[66] Isoyama S Mori S Sugiyama D Kojima Y Tada Y Shitara K. Cancer immunotherapy with PI3K and PD-1 dual-blockade via optimal modulation of T cell activation signal. J Immunother Cancer. (2021) 9:e002279. doi: 10.1136/jitc-2020-002279 34446575 PMC8395371

[67] Sai J Owens P Novitskiy SV Hawkins OE Vilgelm AE Yang J. PI3K inhibition reduces mammary tumor growth and facilitates antitumor immunity and anti-PD1 responses. Clin Cancer Res. (2017) 23:3371–84. doi: 10.1158/1078-0432.CCR-16-2142 PMC5479746 28003307

[68] Spranger S Gajewski TF. Impact of oncogenic pathways on evasion of antitumour immune responses. Nat Rev Cancer. (2018) 18:139–47. doi: 10.1038/nrc.2017.117 PMC6685071 29326431

[69] Xu Y Gao J Su Z Dai X Li Y Liu Y. Downregulation of Hlx closely related to the decreased expressions of T-bet and Runx3 in patients with gastric cancer may be associated with a pathological event leading to the imbalance of Th1/Th2. Clin Dev Immunol. (2012) 2012:949821. doi: 10.1155/2012/949821 23243425 PMC3514004

[70] Yang P Qiu G Wang S Su Z Chen J Wang S. The mutations of Th1 cell-specific T-box transcription factor may be associated with a predominant Th2 phenotype in gastric cancers. Int J Immunogenet. (2010) 37:111–5. doi: 10.1111/j.1744-313X.2010.00899.x 20193034

[71] Liu SJ Tsai JP Shen CR Sher YP Hsieh CL Yeh YC. Induction of a distinct CD8 Tnc17 subset by transforming growth factor-beta and interleukin-6. J Leukoc Biol. (2007) 82:354–60. doi: 10.1189/jlb.0207111 17505023

[72] Zhang Y Zhang Y Gu W Sun B. TH1/TH2 cell differentiation and molecular signals. Adv Exp Med Biol. (2014) 841:15–44. doi: 10.1007/978-94-017-9487-9_2 25261203

[73] Tong Q Liu K Lu XM Shu XG Wang GB. Construction and characterization of a novel fusion protein MG7-scFv/SEB against gastric cancer. J BioMed Biotechnol. (2010) 2010:121094. doi: 10.1155/2010/121094 20339532 PMC2843864

[74] Zhang X Zhang P Cong A Feng Y Chi H Xia Z. Unraveling molecular networks in thymic epithelial tumors: deciphering the unique signatures. Front Immunol. (2023) 14:1264325. doi: 10.3389/fimmu.2023.1264325 37849766 PMC10577431

[75] El-Omar EM Rabkin CS Gammon MD Vaughan TL Risch HA Schoenberg JB. Increased risk of noncardia gastric cancer associated with proinflammatory cytokine gene polymorphisms. Gastroenterology. (2003) 124:1193–201. doi: 10.1016/S0016-5085(03)00157-4 12730860

[76] Zhao CN Xiao LL Zhang Y. Effects of helicobacter pylori infection on the prognosis of chronic atrophic gastritis by inducing the macrophage polarization. Gastroenterology Res. (2023) 16:226–33. doi: 10.14740/gr1636 PMC10482605 37691749

[77] Lu Z Pang T Yin X Cui H Fang G Xue X. Delivery of TSPAN1 siRNA by novel th17 targeted cationic liposomes for gastric cancer intervention. J Pharm Sci. (2020) 109:2854–60. doi: 10.1016/j.xphs.2020.05.018 32497593

[78] Brevi A Cogrossi LL Grazia G Masciovecchio D Impellizzieri D Lacanfora L. Much more than IL-17A: cytokines of the IL-17 family between microbiota and cancer. Front Immunol. (2020) 11:565470. doi: 10.3389/fimmu.2020.565470 33244315 PMC7683804

[79] Vitiello GA Miller G. Targeting the interleukin-17 immune axis for cancer immunotherapy. J Exp Med. (2020) 217:e20190456. doi: 10.1084/jem.20190456 31727783 PMC7037254

[80] Wang JT Li H Zhang H Chen YF Cao YF Li RC. Intratumoral IL17-producing cells infiltration correlate with antitumor immune contexture and improved response to adjuvant chemotherapy in gastric cancer. Ann Oncol. (2019) 30:266–73. doi: 10.1093/annonc/mdy505 30445581

[81] Jiang X Wang J Deng X Xiong F Ge J Xiang B. Role of the tumor microenvironment in PD-L1/PD-1-mediated tumor immune escape. Mol Cancer. (2019) 18:10. doi: 10.1186/s12943-018-0928-4 30646912 PMC6332843

[82] Nelson MA Ngamcherdtrakul W Luoh SW Yantasee W. Prognostic and therapeutic role of tumor-infiltrating lymphocyte subtypes in breast cancer. Cancer Metastasis Rev. (2021) 40:519–36. doi: 10.1007/s10555-021-09968-0 PMC8424653 33963482

[83] Miller TJ Anyaegbu CC Lee-Pullen TF Spalding LJ Platell CF McCoy MJ. PD-L1+ dendritic cells in the tumor microenvironment correlate with good prognosis and CD8+ T cell infiltration in colon cancer. Cancer Sci. (2021) 112:1173–83. doi: 10.1111/cas.v112.3 PMC7935795 33345422

[84] Zhang Y Yu M Jing Y Cheng J Zhang C Cheng L. Baseline immunity and impact of chemotherapy on immune microenvironment in cervical cancer. Br J Cancer. (2021) 124:414–24. doi: 10.1038/s41416-020-01123-w PMC7852680 33087896

[85] Wang YL Gong Y Lv Z Li L Yuan Y. Expression of PD1/PDL1 in gastric cancer at different microsatellite status and its correlation with infiltrating immune cells in the tumor microenvironment. J Cancer. (2021) 12:1698–707. doi: 10.7150/jca.40500 PMC7890312 33613757

[86] Meng X Zhu S Dong Q Zhang S Ma J Zhou C. Expression of Th17/Treg related molecules in gastric cancer tissues. Turk J Gastroenterol. (2018) 29:45–51. doi: 10.5152/tjg.2018.17114 29391307 PMC6322628

[87] Urakawa S Yamasaki M Makino T Kurokawa Y Yamamoto K Goto K. The impact of ICOS(+) regulatory T cells and Helicobacter pylori infection on the prognosis of patients with gastric and colorectal cancer: potential prognostic benefit of pre-operative eradication therapy. Cancer Immunol Immunother. (2021) 70:443–52. doi: 10.1007/s00262-020-02696-4 PMC10992938 32803278

[88] Lu L Barbi J Pan F. The regulation of immune tolerance by FOXP3. Nat Rev Immunol. (2017) 17:703–17. doi: 10.1038/nri.2017.75 PMC5793224 28757603

[89] Ma ES Wang ZX Zhu MQ Zhao J. Immune evasion mechanisms and therapeutic strategies in gastric cancer. World J Gastrointest Oncol. (2022) 14:216–29. doi: 10.4251/wjgo.v14.i1.216 PMC8790417 35116112

[90] Zhou JG Liang R Wang HT Jin SH Hu W Frey B. Identification and characterization of circular RNAs as novel putative biomarkers to predict anti-PD-1 monotherapy response in metastatic melanoma patients - Knowledge from two independent international studies. Neoplasia. (2023) 37:100877. doi: 10.1016/j.neo.2023.100877 36696838 PMC9879779

[91] Ma GF Miao Q Liu YM Gao H Lian JJ Wang YN. High FoxP3 expression in tumour cells predicts better survival in gastric cancer and its role in tumour microenvironment. Br J Cancer. (2014) 110:1552–60. doi: 10.1038/bjc.2014.47 PMC3960619 24548868

[92] Qu Y Wang X Bai S Niu L Zhao G Yao Y. The effects of TNF-α/TNFR2 in regulatory T cells on the microenvironment and progression of gastric cancer. Int J Cancer. (2022) 150:1373–91. doi: 10.1002/ijc.v150.8 PMC9298834 34766338

[93] Wang Z Zhao Y Zhang LJCPA. Emerging trends and hot topics in the application of multi-omics in drug discovery: A bibliometric and visualized study. Current Pharmaceutical Analysis (2024) 21:20-32. doi: 10.1016/j.cpan.2024.12.001

[94] Chen J Lin A Luo PJCPA. Advancing pharmaceutical research: A comprehensive review of cutting-edge tools and technologies. Current Pharmaceutical Analysis (2024) 21:1–19. doi: 10.1016/j.cpan.2024.11.001

[95] Rossi A Belmonte B Carnevale S Liotti A De Rosa V Jaillon S. Stromal and immune cell dynamics in tumor associated tertiary lymphoid structures and anti-tumor immune responses. Front Cell Dev Biol. (2022) 10:933113. doi: 10.3389/fcell.2022.933113 35874810 PMC9304551

[96] Pagès F Berger A Camus M Sanchez-Cabo F Costes A Molidor R. Effector memory T cells, early metastasis, and survival in colorectal cancer. N Engl J Med. (2005) 353:2654–66. doi: 10.1056/NEJMoa051424 16371631

[97] Hsiao YW Lai TC Lin YH Su CY Lee JJ Liao AT. Granulysin expressed in a humanized mouse model induces apoptotic cell death and suppresses tumorigenicity. Oncotarget. (2017) 8:83495–508. doi: 10.18632/oncotarget.11473 PMC5663531 29137359

[98] Cao Z Xu X Luo X Li L Huang B Li X. Role of RANTES and its receptor in gastric cancer metastasis. J Huazhong Univ Sci Technolog Med Sci. (2011) 31:342–7. doi: 10.1007/s11596-011-0378-3 21671175

[99] Zhai X Zhang H Xia Z Liu M Du G Jiang Z. Oxytocin alleviates liver fibrosis via hepatic macrophages. JHEP Rep. (2024) 6:101032. doi: 10.1016/j.jhepr.2024.101032 38882603 PMC11177191

[100] Xiao J Lin H Liu B Xia Z Zhang J Jin J. Decreased S1P and SPHK2 are involved in pancreatic acinar cell injury. Biomark Med. (2019) 13:627–37. doi: 10.2217/bmm-2018-0404 31157539

[101] Xiao J Huang K Lin H Xia Z Zhang J Li D. Mogroside II(E) inhibits digestive enzymes via suppression of interleukin 9/interleukin 9 receptor signalling in acute pancreatitis. Front Pharmacol. (2020) 11:859. doi: 10.3389/fphar.2020.00859 32587518 PMC7298197

[102] Zhang H Xia T Xia Z Zhou H Li Z Wang W. KIF18A inactivates hepatic stellate cells and alleviates liver fibrosis through the TTC3/Akt/mTOR pathway. Cell Mol Life Sci. (2024) 81:96. doi: 10.1007/s00018-024-05114-5 38372748 PMC10876760

[103] Wang JF Wang JS Liu Y Ji B Ding BC Wang YX. Knockdown of integrin beta1 inhibits proliferation and promotes apoptosis in bladder cancer cells. Biofactors. (2025) 51:e2150. doi: 10.1002/biof.2150 39644117

[104] Hou PF Zhu LJ Chen XY Qiu ZQ. Age-related changes in CD4+CD25+FOXP3+ regulatory T cells and their relationship with lung cancer. PloS One. (2017) 12:e0173048. doi: 10.1371/journal.pone.0173048 28253320 PMC5333862

[105] Liu C Workman CJ Vignali DA. Targeting regulatory T cells in tumors. FEBS J. (2016) 283:2731–48. doi: 10.1111/febs.2016.283.issue-14 26787424

[106] Lee HE Chae SW Lee YJ Kim MA Lee HS Lee BL. Prognostic implications of type and density of tumour-infiltrating lymphocytes in gastric cancer. Br J Cancer. (2008) 99:1704–11. doi: 10.1038/sj.bjc.6604738 PMC2584941 18941457

[107] Hu G Wang S. Tumor-infiltrating CD45RO(+) memory T lymphocytes predict favorable clinical outcome in solid tumors. Sci Rep. (2017) 7:10376. doi: 10.1038/s41598-017-11122-2 28871164 PMC5583330

[108] Gu J Wang Y Zhang H Gu H Zhu H. SIGLEC1 has the potential to be an immune-related prognostic indicator in colon adenocarcinoma: a study based on transcriptomic data and Mendelian randomization analysis. Discov Oncol. (2025) 16:324. doi: 10.1007/s12672-025-02093-2 40088346 PMC11910455

[109] Wakatsuki K Sho M Yamato I Takayama T Matsumoto S Tanaka T. Clinical impact of tumor-infiltrating CD45RO^+^ memory T cells on human gastric cancer. Oncol Rep. (2013) 29:1756–62. doi: 10.3892/or.2013.2302 23440298

[110] Xie H Xi X Lei T Liu H Xia Z. CD8(+) T cell exhaustion in the tumor microenvironment of breast cancer. Front Immunol. (2024) 15:1507283. doi: 10.3389/fimmu.2024.1507283 39717767 PMC11663851

[111] Zhang J Peng G Chi H Yang J Xie X Song G. CD8 + T-cell marker genes reveal different immune subtypes of oral lichen planus by integrating single-cell RNA-seq and bulk RNA-sequencing. BMC Health. (2023) 23:464. doi: 10.1186/s12903-023-03138-0 PMC10329325 37422617

[112] Xia Z Chen S He M Li B Deng Y Yi L. Editorial: Targeting metabolism to activate T cells and enhance the efficacy of checkpoint blockade immunotherapy in solid tumors. Front Immunol. (2023) 14:1247178. doi: 10.3389/fimmu.2023.1247178 37575246 PMC10415066

[113] Xiong J Chi H Yang G Zhao S Zhang J Tran LJ. Revolutionizing anti-tumor therapy: unleashing the potential of B cell-derived exosomes. Front Immunol. (2023) 14:1188760. doi: 10.3389/fimmu.2023.1188760 37342327 PMC10277631

[114] Wang Y Wang J Liu J Zhu H. Immune-related diagnostic markers for benign prostatic hyperplasia and their potential as drug targets. Front Immunol. (2024) 15:1516362. doi: 10.3389/fimmu.2024.1516362 39703506 PMC11655502

[115] Chi H Zhao S Yang J Gao X Peng G Zhang J. T-cell exhaustion signatures characterize the immune landscape and predict HCC prognosis via integrating single-cell RNA-seq and bulk RNA-sequencing. Front Immunol. (2023) 14:1137025. doi: 10.3389/fimmu.2023.1137025 37006257 PMC10050519

[116] Xue M Jin W. Editorial: Immunological precision therapeutics: integrating multi-omics technologies and comprehensive approaches for personalized immune intervention. Front Immunol. (2025) 16:1581238. doi: 10.3389/fimmu.2025.1581238 40114915 PMC11922912

[117] Huang L Sun F Liu Z Jin W Zhang Y Chen J. Probing the potential of defense response-associated genes for predicting the progression, prognosis, and immune microenvironment of osteosarcoma. Cancers (Basel). (2023) 15:2405. doi: 10.3390/cancers15082405 37190333 PMC10137316

[118] Wang Y Zhu C Song W Li J Zhao G Cao H. PD-L1 expression and CD8(+) T cell infiltration predict a favorable prognosis in advanced gastric cancer. J Immunol Res. (2018) 2018:4180517. doi: 10.1155/2018/4180517 30003113 PMC5996418

[119] Thompson ED Zahurak M Murphy A Cornish T Cuka N Abdelfatah E. Patterns of PD-L1 expression and CD8 T cell infiltration in gastric adenocarcinomas and associated immune stroma. Gut. (2017) 66:794–801. doi: 10.1136/gutjnl-2015-310839 26801886 PMC4958028

[120] Xing X Guo J Ding G Li B Dong B Feng Q. Analysis of PD1, PDL1, PDL2 expression and T cells infiltration in 1014 gastric cancer patients. Oncoimmunology. (2018) 7:e1356144. doi: 10.1080/2162402X.2017.1356144 29399387 PMC5790386

[121] Li Z Jiang Y Li B Han Z Shen J Xia Y. Development and validation of a machine learning model for detection and classification of tertiary lymphoid structures in gastrointestinal cancers. JAMA Netw Open. (2023) 6:e2252553. doi: 10.1001/jamanetworkopen.2022.52553 36692877 PMC10408275

[122] Zhang X Ren B Liu B Wang R Li S Zhao Y. Single-cell RNA sequencing and spatial transcriptomics reveal the heterogeneity and intercellular communication of cancer-associated fibroblasts in gastric cancer. J Transl Med. (2025) 23:344. doi: 10.1186/s12967-025-06376-8 40102930 PMC11917039

[123] Huang RJ Wichmann IA Su A Sathe A Shum MV Grimes SM. A spatial transcriptomic signature of 26 genes resolved at single-cell resolution characterizes high-risk gastric cancer precursors. NPJ Precis Oncol. (2025) 9:52. doi: 10.1038/s41698-025-00816-w 40000871 PMC11861308

